# Crossing boundaries: improving communication in cerebral palsy care

**Published:** 2012-03-30

**Authors:** Jitske Gulmans

**Affiliations:** Hanzehogeschool Groningen, CaRES Centre of Applied Research & Innovation (www.hanze.nl/kenniscentrumcares), P.O. Box 70030, 9700 RM Groningen, The Netherlands.E-mail: j.gulmans@pl.hanze.nl

## Background

In the Netherlands, children with cerebral palsy are the largest diagnostic group treated in paediatric rehabilitation, requiring specialized health-, education- and social services of multiple professionals from diverse organizations. In order to provide integrated care in these settings, effective care coordination is essential, though in practice this is often challenged by inadequate communication across the child’s care network.

## Objective

In order to improve communication across the integrated care setting of cerebral palsy, the present study aimed to (i) identify experienced gaps in parent-professional and inter-professional communication in three Dutch cerebral palsy care regions; and (ii) obtain insight in the feasibility and usability of an eHealth-application as a potential improvement strategy for the identified gaps in communication in each care region.

## Methods

In view of the shortcomings of available methodology to evaluate patient care communication in integrated care settings such as cerebral palsy, we developed a mixed method approach attuned to the multiple communication links and evaluation perspectives inherent to integrated care settings and based on relevant quality dimensions specified by the Institute of Medicine and the Chronic Care Model. This approach was subsequently applied in three Dutch cerebral palsy care regions in order to identify experienced gaps in communication relevant to both parents and involved professionals. The identified gaps and needs of improvement were translated into functional specifications and technical requirements of an asynchronous secure web-based system for parent-professional and inter-professional communication, which was developed in an iterative design process and subsequently evaluated in a 6-months pilot in each care region.

## Results

Parents primarily experienced gaps in inter-professional communication, ranging from lack of cooperation and patient-centeredness to insufficient information-exchange and consistency of information. Consequently, parents had to take up the role of messenger of information and/or care coordinator [http://www.ijic.org/index.php/ijic/article/view/672]. Involved professionals recognized these gaps and primarily attributed them to capacity problems, lack of interdisciplinary guidelines and a clear definition of roles, tasks and responsibilities. Based on these gaps an asynchronous secure web-based system for parent-professional and inter-professional communication was developed, aimed to increase patient centeredness, facilitate inter-professional contact and enhance network transparency. A 6-month pilot evaluation in each care region showed broad variation in frequency of system use and its experienced added value, which was related to users’ actual consultation needs and the complexity of the child’s care network.

## Conclusions relevant for integrated care

The system’s technical feasibility and added value, although more pronounced for parents than professionals, merit further research into its effective implementation for those patients who require frequent consultation across various organizations and settings. In addition, further development of the technology should focus on incorporating advanced consultation options, next to increasing the system’s ease of use and its integration into daily care practice. This requires an interactive process of co-creation with relevant stakeholders, in order to realize a ‘fit-for-all’ service tailored to specific user needs.

With respect to the methodology to evaluate patient care communication across integrated care settings like cerebral palsy, the mixed method approach worked as a “funnel” which proved to be useful to identify relevant gaps from both the perspective of parents as well as involved professionals. In addition to relevant quality frameworks we applied in this study, further research could focus on clarifying the role of patient care communication in realizing informational, relational and management continuity of care. Such knowledge could guide short and longer-term efforts to improve parent-professional and inter-professional communication in the care of children with special health care needs.

## Figures and Tables

**Figure 1.**
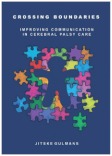
Titel page thesis.

